# Origin and Evolution of Cracks in the Glaze Surface of a Ceramic during the Cooling Process

**DOI:** 10.3390/ma16165508

**Published:** 2023-08-08

**Authors:** Tiantian Chen, Bin Gong, Chun’an Tang

**Affiliations:** 1 School of Resources and Civil Engineering, Northeastern University, Shenyang 110819, China; chentiantian0304@163.com; 2 Department of Civil and Environmental Engineering, Brunel University London, London UB8 3PH, UK; 3 State Key Laboratory of Coastal and Offshore Engineering, Dalian University of Technology, Dalian 116024, China; tca@mail.neu.edu.cn

**Keywords:** ceramic, craquelure, glaze cracking, crack propagation, cooling

## Abstract

Because of the significant difference between the thermal expansion coefficients of ceramic blank and glaze, the glaze typically undergoes more pronounced shrinkage than the blank during ceramic cooling, which results in high stress concentrations and cracking. In this study, the mechanical mechanism of glaze cracking is studied, based on the statistical strength theory, damage mechanics, and continuum mechanics. Furthermore, the influence of the glaze layer thickness, heat transfer coefficient, expansion coefficient, and temperature difference on the creation and propagation of inner microcracks is systematically investigated, and the final discrete fracture network of ceramics is discussed at the specific crack saturation state. The results show that (1) a higher heat transfer coefficient will lead to a more uniform distribution of the surface temperature and a faster cooling process of the ceramics, reducing the number of microcracks when the ambient temperature is reached; (2) the thinner glaze layer is less prone to cracking when its thickness is smaller than that of the blank. However, when the thickness of the glaze layer is similar to that of the blank, the increased thickness of the glaze layer will increase the number of cracks on its surface; and (3) when the expansion coefficient of the glaze layer is smaller than that of the blank, cracks will not occur inside the glaze layer. However, as the coefficient of the thermal expansion of the glaze layer continuously rises, the number of cracks on its surface will first increase and then decrease.

## 1. Introduction

Ceramic products are very common in daily life because of their diverse shapes, bright colors, ease of cleaning, stable mechanical performance, and adequate industrial capabilities. However, due to the mechanical behaviors of ceramic glaze under external loads, craquelures may occur during the process of ceramic production and use. On the one hand, the formation of craquelures will affect product life and cause economic losses. On the other hand, some cracked porcelains are favored by consumers because of their attractive fracture networks and artistic beauty.

**Figure 1:**
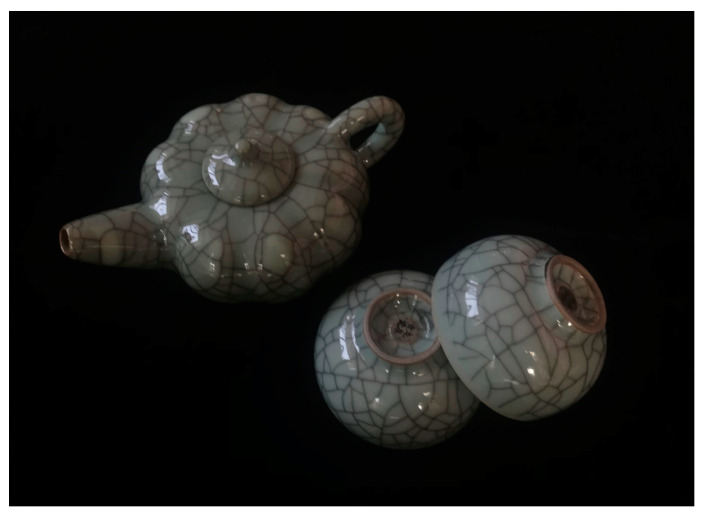
The craquelures on a ceramic surface.

The craquelures on ceramic surfaces are widely observed; for example, the cracked teapot shown in Figure 1 exhibits this feature. Actually, these cracks often appear on the surface of multi-layer media [1,2,3]. Under the combined effects of high temperature, high humidity, and high internal stress, the deformation difference between the surface medium and the internal medium will lead to high stress concentrations and the initiation of microcracks [4,5,6]. Then, the formed cracks gradually expand and penetrate each other until craquelures form. Depending upon the various material properties and environmental factors, many different kinds of craquelures may occur. The formation mechanism of such discrete fracture networks has attracted widespread attention [7,8,9,10]. Hot ceramics may generate high thermal stresses during the cooling process, resulting in severe damage and affecting the mechanical responses of ceramics, according to the results of previous studies [11,12,13,14,15]. High temperature is the main factor affecting the performance of ceramics [16,17,18]. Meanwhile, temperature variations can weaken ceramic structures and make them more prone to cracking. Moreover, inherent defects like pores, inclusions, or grain boundaries act as stress concentrators, making ceramics more susceptible to cracking. Researchers have investigated the different crack growth mechanisms, such as intergranular, transgranular, or mixed-mode cracking, to understand how cracks propagate through ceramic materials, examining fractured ceramics to analyze crack patterns, origins, and failure modes, providing insights into their underlying causes [19,20,21]. The various methods to mitigate cracks, including improving material properties, optimizing manufacturing processes, and implementing stress-relieving techniques like annealing or thermal cycling, have also been explored [22,23,24].

However, the evolution mechanisms of surface cracks in multi-layered quasi-brittle materials, such as ceramic structures, remain unclear. The possible cause of ceramic cracking is the concentrated high stresses induced by the different thermal expansion coefficients of ceramic blank and glaze. When the thermal expansion coefficient of the glaze is greater than that of the blank, and the shrinkage of the glaze will be more significant than that of the blank during cooling [25,26,27,28]. When the stress buildup exceeds the ultimate strength of the glaze layer, the cracking phenomenon will occur. In fact, a number of ice-cracked ceramics were produced in Ancient China, and many of them are precious works of art. Thus, several researchers have investigated the manufacturing process and control factors of these fracture networks [29,30,31,32]. But, the current studies mainly focus on the qualitative analysis of the cracking phenomenon, and the quantitative analysis of the control factors is still lacking.

In this study, the formation mechanism of the discrete fracture network on ceramic surfaces is investigated to improve our understanding of the progressive creation and development of microcracks in the glaze layer, affected by the gradual process of stress concentration, stress release, and stress transfer. Based on the statistical strength theory, damage mechanics, and continuum mechanics, the ceramic models including blank and glaze are established, and the initiation, propagation, and saturation process of cracks in the glaze layer is modeled. Furthermore, the geometric and physical parameters of the glaze layer during the cooling process are studied, including glaze thickness, expansion coefficients, heat transfer coefficients, etc. Additionally, the preventive and control measures for craquelures are analyzed, and guidance for avoiding material waste during the production of ceramic products is discussed.

## 2. Materials and Methods

### 2.1. Numerical Method

#### 2.1.1. Constitutive Relation

In this study, the three-dimensional thermal-mechanical coupled realistic fracture process analysis (RFPA) method [33,34] is used. RFPA is a finite element method (FEM) based software which is developed according to the statistical strength theory, damage mechanics, and continuum mechanics, and has been used by many researchers to simulate the generation and development of cracks in quasi-brittle materials. The main characteristic of RFPA is to consider the influence of material heterogeneity on non-linear mechanical behaviors, and the related physical and mechanical parameters are assigned to obey the Weibull distribution function [35,36], shown as follows:

(1) $$f\left(u\right)=\frac{m}{u_{0}}{\left(\frac{u}{u_{0}}\right)}^{m-1}e^{-{\left(\frac{u}{u_{0}}\right)}^{m}}$$

where $f\left(u\right)$ is the probability density function of the relevant material parameters; u is the specific parameter, such as strength, elastic modulus, and thermal expansion coefficient; $u_{0}$ is the average value of the parameter u; *m* is the heterogeneity index within the range of (1, +∞), and the higher the *m*, the more uniform the material.

**Figure 2:**
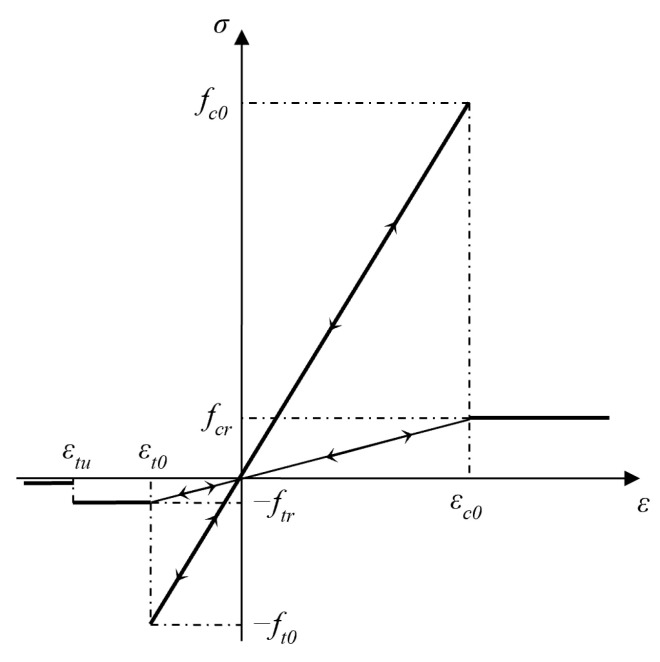
Damage constitutive law of each element.

In Figure 2, the stress–strain relationship of mesoscopic elements under a uniaxial stress state is shown in RFPA. When the stress state of an element reaches the failure criterion provided by the user, the software will determine that the element is damaged at the current time step. Meanwhile, the degradation of the element elastic modulus during the loading process can be determined according to the following equation:

(2) $$E=(1-\omega )E_{0}$$

where E is the elastic modulus value of the damaged element; $E_{0}$ is the initial value of the elastic modulus; and ω is the damage variable.

When loading to a certain extent, the compression-shear stress state of an element may reach the Mohr–Coulomb criterion. At this moment, the damage variable ω is:

(3) $$\omega =\left\{\begin{array}{c} 0\\ 1-\frac{{\lambda \varepsilon }_{t0}}{\varepsilon }\\ 1 \end{array}\right.\begin{array}{c} \varepsilon >\varepsilon_{t0}\\ \varepsilon_{tu}<\varepsilon \leq \varepsilon_{t0}\\ \varepsilon \leq \varepsilon_{tu} \end{array}$$

where λ is the residual strength coefficient, which can be calculated using $\lambda =f_{tr}/f_{t0}$; $\varepsilon_{t0}$ is the compressive strain threshold; and $\varepsilon_{tu}$ is the ultimate compressive strain

When the suffered tensile stress of an element satisfies the maximum tensile stress criterion, tensile damage will occur, and the corresponding damage variable ω is:

(4) $$\omega =\left\{\begin{array}{c} 0\\ 1-\frac{\lambda \varepsilon_{c0}}{\varepsilon } \end{array}\begin{array}{c} \varepsilon <\varepsilon_{c0}\\ \varepsilon \geq \varepsilon_{c0} \end{array}\right.$$

where $\varepsilon_{c0}$ is the tensile strain threshold.

#### 2.1.2. Temperature Equation

The thermo-mechanical coupling is controlled by the following equations [37,38,39]:

(5) $$kT_{ii}+Q=\rho c\dot{T}+\beta T_{0}\dot{\varepsilon_{kk}}$$

(6) $$\sigma_{ij,ii}+F_{bi}=\rho \ddot{u_{i}}$$

(7) $$\varepsilon_{ij}=(u_{i,j}+u_{j,i})/2$$

(8) $$\sigma_{ij}=\lambda \varepsilon_{mm}\delta_{ij}+2G\varepsilon_{ij}-\beta \Delta T\delta_{ij}$$

(9) β=(3λ+2G)α

where $\sigma_{ij}$ is the stress term; $\varepsilon_{ij}$ is the strain term; $F_{bi}$ is the mass force; $\ddot{u_{i}}$ is the inertial variable; β is the thermal stress coefficient; ΔT is the temperatures difference, i.e., $\Delta T=T-T_{0}$; $T_{0}$ is the initial temperature; $\delta_{ij}$ is the Kronecker function; Q is the heat generation; λ is the Lame constant; G is the shear modulus; α is the thermal expansion coefficient; k is the thermal conductivity; ρ is the density; and c is the specific heat.

The heat conduction equations [37,38,39] are as follows:

(10) $$k_{x}\frac{\partial^{2}T}{\partial x^{2}}+k_{y}\frac{\partial^{2}T}{\partial y^{2}}+k_{z}\frac{\partial^{2}T}{\partial z^{2}}+Q=\rho c\frac{\partial T}{\partial t}\;(\mathrm{On}\;\Omega^{e})$$

(11) $${T\left(P,t\right)|}_{P\epsilon S_{1}}=\varphi (P,t)\;(\mathrm{The}\;\mathrm{boundary}\;\mathrm{condition}\;\mathrm{is}\;S_{1}^{e}.)$$

(12) $${-k_{n}\frac{\partial T}{\partial n}|}_{P\epsilon S_{2}}=\bar{q_{S_{2}}}\;(\mathrm{The}\;\mathrm{boundary}\;\mathrm{condition}\;\mathrm{is}\;S_{2}^{e}.)$$

(13) $${k_{n}\frac{\partial T}{\partial n}|}_{P\epsilon S_{3}}=h(T_{s}-T)\;(\mathrm{The}\;\mathrm{boundary}\;\mathrm{condition}\;\mathrm{is}\;S_{3}^{e}.)$$

(14) $${T|}_{t=t_{0}}=T(P,t_{0})\;(\mathrm{at}\;\mathrm{time}\;t_{0}^{e})$$

The equilibrium equations and boundary conditions are as follows:

(15) $$\sigma_{ij,j}+F_{bi}=0\;(\mathrm{balance}\;\mathrm{equation})$$

(16) $$\varepsilon_{ij}=(u_{i,j}+u_{j,i})/2\;(\mathrm{geometrical}\;\mathrm{equation})$$

(17) $$\sigma_{ij}=\lambda \varepsilon_{mm}\delta_{ij}+2G\varepsilon_{ij}-\beta \Delta T\delta_{ij}\;(\mathrm{physics}\;\mathrm{equation})$$

(18) $$f_{i}=\tilde{f_{i}}\;(\mathrm{force}\;\mathrm{boundary}\;\mathrm{condition})$$

(19) $$u_{i}=\tilde{u_{i}}\;(\mathrm{displacement}\;\mathrm{boundary}\;\mathrm{condition})$$

(20) β=(3λ+2G)α (displacement boundary condition)

where $k_{x}$, $k_{y}$, and $k_{z}$ are the heat conduction coefficients of the element along the *x*, *y*, and *z* directions, respectively; $S_{1}^{e}$, $S_{2}^{e}$, and $S_{3}^{e}$ are the first, second, and third thermal boundary conditions, respectively; $t_{0}^{e}$ is the initial moment; φ(P,t) is the distribution function of temperature on the boundary point *P* at the time *t*; $\bar{q_{S_{2}}}$ is the boundary heat flow density; *h* is the heat transfer coefficient between the boundary and the environment; $T_{s}$ is the ambient temperature; $\Omega^{e}$ is the entire solution domain of the element; $\tilde{f_{i}}$ is the stress boundary; and $\tilde{u_{i}}$ is the displacement boundary.

### 2.2. Model Configuration

In this study, two kinds of materials are considered, i.e., glaze and blank. The formation process of cracks inside the glaze layer during the cooling process is simulated under the condition of cooling from 1230 °C [24]. The influence of the layer thickness, expansion coefficient, and heat transfer coefficient are investigated. For quantitatively analyzing the simulated image data, the Crack Image Analysis System (CIAS) software developed by the research team led by Prof. Tang [40,41] at Nanjing University, China, (www.climate-engeo.com (accessed on 10 January 2023)) is used. The fractal dimension value can be directly obtained through CIAS based on the area perimeter method, which can be expressed as follows:

(21) $$\log\left(P\right)=D/2\times log\;(A)+C\;(2-3)$$

where *P* represents the perimeter of one block identified on the given crack image; *A* represents the area of the corresponding block; *C* is the computing constant; and *D* is the fractal dimension of the related block shown on the crack image.

The model with the size of 100 m × 100 mm × (2*h_g_* + *h_b_*), where *h_g_* = 0.5 mm, 1 mm, 1.5 mm, 2 mm, 2.5 mm, 3 mm, and *h_b_* = 2 mm, is established. The number of model elements is 300 × 300 × 3 × (2*h′_g_* + *h′_b_*) (*h′_g_* = 0.5, 1, 1.5, 2, 2.5, 3, and *h′_b_* = 2). The blank size is (100 × *h_g_*) mm × (100 × *h_g_*) mm × 2 mm, where *h_g_* is the glaze thickness; and *h_b_* is the blank thickness. The glaze layer completely encloses the blank. The physical and mechanical parameters of the model materials shown in Table 1 are determined by referring to [24] and identifying the numerical trials. The model is calibrated by comparing the related results with those in the literature [15,24] in terms of the strength of the materials and the morphology and spatial distribution of the cracks.

**Figure 3:**
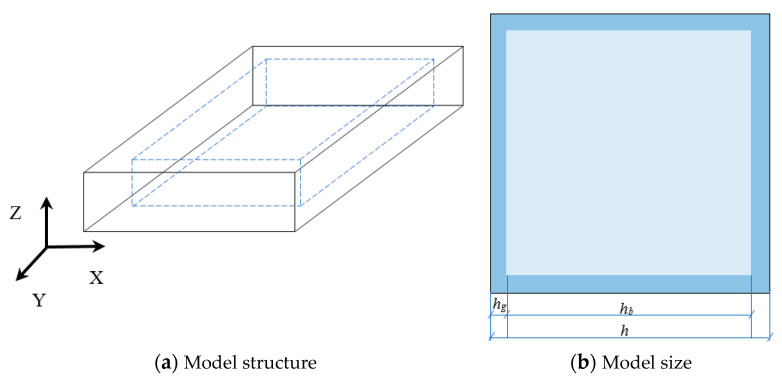
Diagram of the model.

Figure 3 shows the model geometry. The model is established to simulate the behaviors of the ceramic sheet in the kiln. The model is fixed at the bottom, and the other surfaces are free. The third type of temperature loading is used, and the ambient temperature is set at 20 °C. After firing to 1230 °C, the creation and development of cracks inside the ceramic sheet during cooling under different conditions are observed.

On the one hand, the fracture modes of the material layers are affected by their geometry. On the other hand, the cracking of the glaze layer is influenced by the physical and mechanical parameters. Moreover, the heat exchange rate between the ceramic plate and the external environment also affects the appearance of craquelure. To take these factors into account, different glaze thicknesses are set, i.e., *h_g_* = 0.5 mm, 1 mm, 1.5 mm, and 2 mm, 2.5 mm, 3 mm; different coefficient of thermal expansion (CTE) of a glaze are used, i.e., *α_g_* = 5.5 × 10^−6^/K, 5.75 × 10^−6^/K, 6 × 10^−6^/K, 6.25 × 10^−6^/K, 6.5 × 10^−6^/K, and 6.75 × 10^−6^/K; and different heat transfer coefficients are applied, i.e., *β* = 10 W/(m^2^·K), 25 W/(m^2^·K), 50 W/(m^2^·K), 100 W/(m^2^·K), 150 W/(m^2^·K), and 200 W/(m^2^·K).

## 3. Results

### 3.1. The Influence of Temperature Difference on the Fracture Process

Due to the different cooling rates, the heat dissipation speed of ceramic materials is also different. The surface of ceramics will produce a temperature change trend, leading to various modes of fracture development. In this section, the heat transfer coefficient *β* = 10 W/(m^2^·K) (slow cooling) and *β* = 100 W/(m^2^·K) (fast cooling) are taken into account.

#### 3.1.1. The Fracture Formation under Slow Cooling

**Figure 4:**
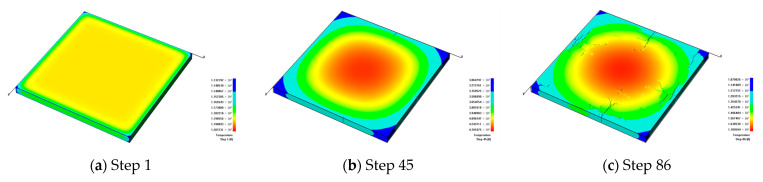

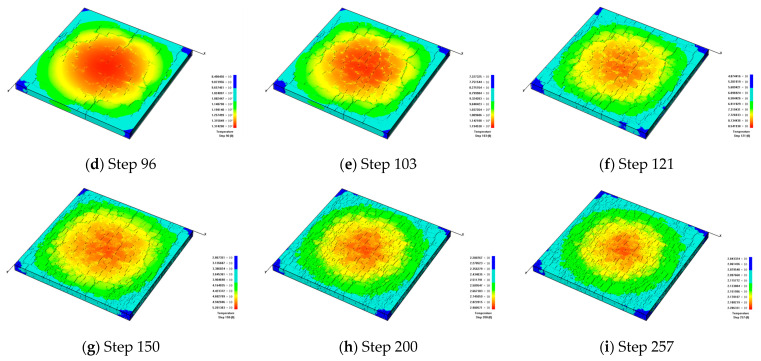
The formation process of glaze fractures in ceramics during slow cooling when *h_g_* = 2 mm, *β* = 10 W/(m^2^·K) and *α_g_* = 6/K, presented by temperature contours.

Figure 4 shows that the model gradually cools down to about 300 °C with a slow cooling rate. From Figure 4a,b, it can be seen that because there is no constraint around the surrounding boundaries under the effect of cold shrinkage, fractures start to sprout around the model. Figure 4c indicates that due to the thin thickness of the model, fractures first generate at the weak areas and then extend to the upper and lower surfaces of the model. Meanwhile, the directions of the generated fractures are mainly perpendicular to the model boundaries.

As the temperature continues to decrease, fractures begin to expand on the surface of the glaze layer. Because of the slow cooling rate, there is a noticeable temperature difference between the environmental temperature and the internal temperature of the model. During heat dissipation and contraction, the temperature of the model decreases sequentially from the outside to the inside, and the temperature difference between the outside and the inside causes fractures. Currently, the formed fractures are mainly parallel to the model boundaries. In this case, the horizontal and vertical fractures gradually intersect and finally form a discrete fracture network, i.e., craquelures, as shown in Figure 4d,e. With the decreasing temperature, the cold shrinkage process continues. At the same time, the outer fractures in the model begin to be intensified, as shown in Figure 4f,g. As the model continues to cool down, the internal fractures also appear to be intensified, as shown in Figure 4h. After cooling to a certain degree, the surface temperature distribution of the model becomes relatively uniform and similar to the environmental environment. At this moment, since there is no noticeable temperature difference, few new cracks will appear, and the crack situation on the model surface tends to become stable, as shown in Figure 4i.

#### 3.1.2. The Fracture Formation under Fast Cooling

**Figure 5:**
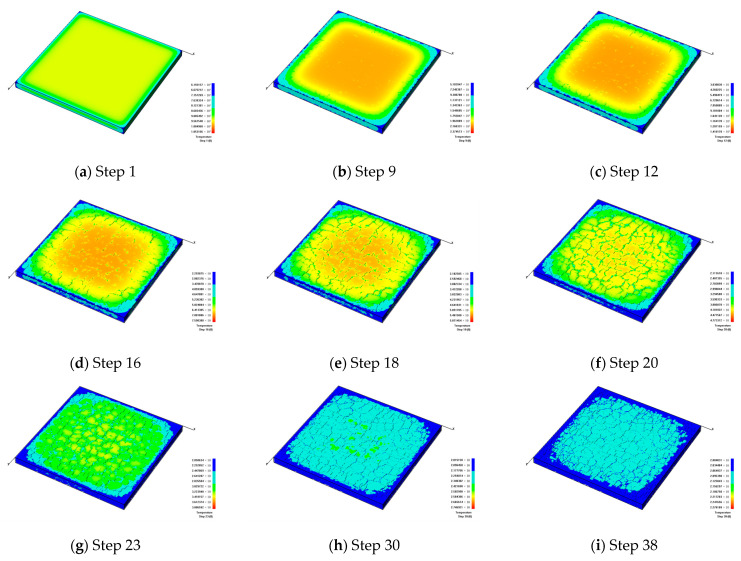
The formation process of glaze fractures in ceramics during fast cooling when *h_g_* = 2 mm, *β* = 100 W/ (m^2^·K), and *α_g_* = 6 × 10^−6^/K, presented by temperature contours.

Figure 5 shows the fracture evolution of ceramic glaze during the fast cooling process. The model is only constrained at the bottom, and the outer parts of the model are first cooled during the rapid cooling. The temperatures of the outer parts of the model are obviously different from those of the internal parts, forming high inner temperature stresses, as shown in Figure 5a,b. Because the glaze layer is on the surface of the ceramics and the glaze layer cools faster, such cooling causes the glaze layer to shrink dramatically. Under the constraint of the blank, high tensile stress concentrations occur on the surface of the glaze layer. As the temperature decreases, the tensile stress accumulated on the surface quickly increases, and new cracks are generated. At the early cooling stage, micro-cracks on the model surface are mainly generated and distributed at the outer parts of the model, as shown in Figure 5c. As the temperature decreases, the cracks propagate towards the inner parts of the model, as shown in Figure 5d,e, and then gradually gather to form craquelures, as shown in Figure 5f,g. Then, the large blocks are divided into small blocks. After the temperature distribution reaches the ambient temperature, few new cracks are generated, as shown in Figure 5h,i. The obvious differences from when compared to the results of slow cooling are that the propagation speed of the fractures is relatively rapid under fast cooling, the effect of the temperature difference is relatively small, and the size of the blocks divided by fractures on the model surface is larger and more uniform.

### 3.2. The Influence of the Glaze Heat Transfer Coefficient

**Figure 6:**
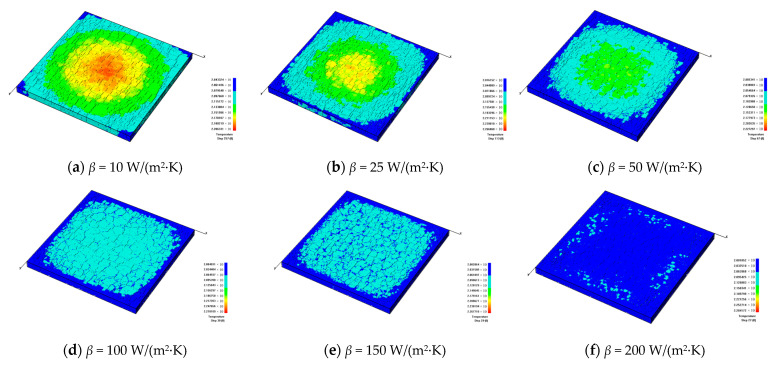
The development of cracks on the glaze surface and the temperature distributions when the glaze layers with different heat transfer coefficients are cooled to the same degree, *h_g_* = 2 mm and *α_g_* = 6 × 10^−6^/K.

To understand the significant influence of the cooling rate on the formation of cracks, the different heat transfer coefficients are investigated, i.e., *β* = 10 W/(m^2^·K), 25 W/(m^2^·K), 50 W/(m^2^·K), 100 W/(m^2^·K), 150 W/(m^2^·K), and 200 W/(m^2^·K). It can be seen from Figure 6 that when the heat transfer coefficient becomes larger, the cooling speed of the model will become faster, and it is less likely to form a significant temperature difference on the model surface. Figure 6 shows that when the models have cooled to the same degree, the larger heat transfer coefficient will lead to a more uniform surface temperature distribution; the larger heat transfer coefficient will cause a more rapid temperature drop, a smaller temperature difference inside the model, and a smaller number of fractures.

**Figure 7:**
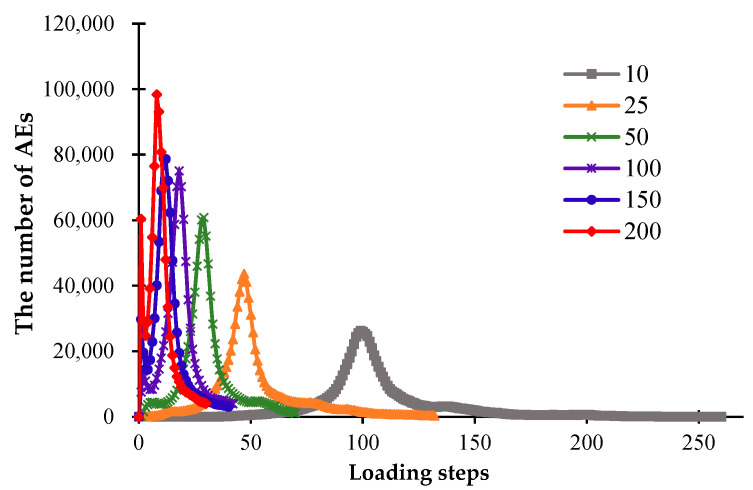
The acoustic emission curves affected by different heat transfer coefficients.

During the fracture evolution of ceramics, the release of elastic energy causing the formation of fractures can occur in the form of acoustic emission (AE). In this study, the AE events produced during the simulations can be modeled by the RFPA code. Figure 7 shows that when *β* = 10 W/(m^2^·K), the model cools down slowly, and the peak of AE events appears late. During the cooling progress, the AEs increase gradually, and the appearance of the peak of AE events mean that the model is completely broken. As the heat transfer coefficient increases, the AE peak appears earlier, and the model cools down to the ambient temperature more quickly. When the heat transfer coefficient reaches more than 50 W/(m^2^·K), the cooling speed of the models becomes faster, and due to the rapid cooling, a large amount of AE events will be generated at the early stage of cooling, and the peak of the AE curve will appear earlier. Simultaneously, a larger heat transfer coefficient will lead to a larger number of AE events, as shown in Figure 7.

**Figure 8:**
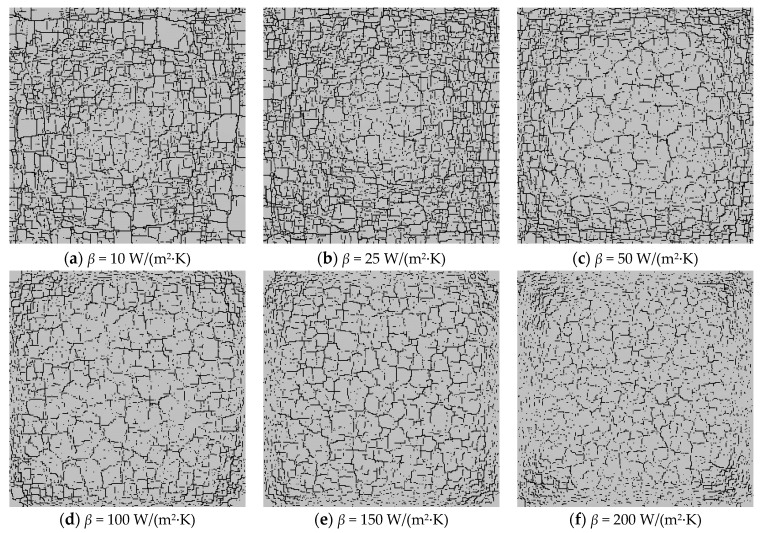
The crack distribution on the glaze surface, affected by different heat transfer coefficients.

**Figure 9:**
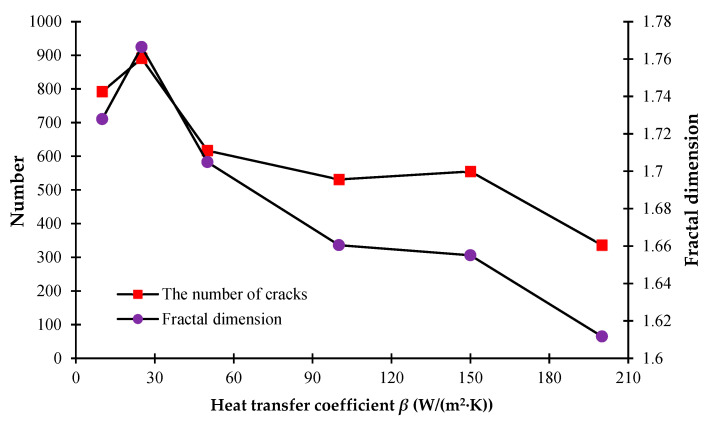
The number of cracks and the fractal dimension curves under different heat transfer coefficients.

Figure 8 shows the simulated distributions of the glaze layers with different heat transfer coefficients, from which we can see that the number of cracks forming inside the glaze layers basically increases with the growth of the heat transfer coefficient. To quantitatively analyze the geometric characteristics of the fracture networks, the CIAS is applied, and the total number of cracks and fractal dimension are shown in Figure 9.

From Figure 9, we can see that with the growth of the heat transfer coefficient, the number of cracks on the model surface basically shows a decreasing trend. However, the number of cracks when *β* = 25 W/ (m^2^·K) is slightly higher than the number when *β* = 10 W/(m^2^·K) because there more large blocks form when *β* = 10 W/(m^2^·K) due to the material inhomogeneity. However, the degree of damage of model materials with *β* = 25 W/(m^2^·K) is greater than that when *β* = 10 W/(m^2^·K). When the heat transfer coefficient is relatively larger, there are more areas of serious damage in the model because of the rapid cooling. The fractal dimension indicates the complexity of the discrete fracture network. The larger the fractal dimension, the more complex the fracture network. From Figure 9, it can also be seen that the fractal dimension basically shows a decreasing trend with the increasing heat transfer coefficient.

### 3.3. The Influence of Glaze Thickness

The thickness of the glaze layer has a significant influence on the discrete fracture network on ceramic surfaces. The thickness of the glaze layer will change the process of stress buildup, stress release, and stress transfer. Thus, appropriately reducing the thickness of the glaze layer can alleviate the formation of cracks. In ice-cracked ceramics, a proper thickness of the glaze layer can lead to a desired fracture network. In this section, the cooling simulation is carried out on the ceramic models with different glaze thicknesses. The creation and evolution of glaze cracks are simulated. To reveal the effect mechanism of glaze thickness on cracking, six thicknesses are taken into account, i.e., *h_g_* = 0.5 mm, 1 mm, 1.5 mm, 2 mm, 2.5 mm, and 3 mm. Moreover, the blank thickness *h_b_* is set at 2 mm.

**Figure 10:**
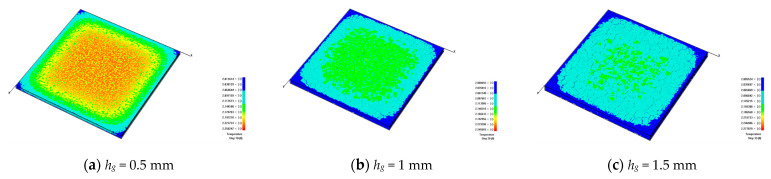

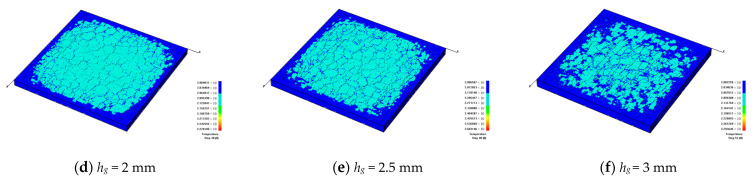
The development of cracks on the glaze surface and the temperature distributions noted when the glaze layers with different glaze thicknesses are cooled to the same degree: *β* = 100 W/(m^2^·K), and *α_g_* = 6 × 10^−6^/K.

Figure 10 shows that when the model temperature drops to the ambient temperature, the cracking degree of the model will rise with the increase in the thickness of the glaze layer. From Figure 10a, it can be seen that when the thickness of the glaze layer *h_g_* = 0.5 mm, the temperature distribution at the outer parts of the glaze layer is lower than that in the middle part, showing a particular gradient change. Figure 10b shows that when *h_g_* = 1 mm, the scattered microcracks appear on the surface of the glaze layer. Because of the generation of microcracks, the heat dissipation rate in the middle of the glaze layer is accelerated, and the temperature difference between the middle and the outer parts of the glaze layer begins to decrease. Figure 10c indicates that when *h_g_* = 1.5 mm, penetrating cracks begin to appear. Simultaneously, compared with the thinner glaze layers, the glaze layer with *h_g_* = 1.5 mm has a greater degree of heat dissipation, and the corresponding surface temperature tends to be uniform. Figure 10d shows that when *h_g_* = 2 mm, the number of through-cracks increases, and the cracks inside the glaze layer become dense. Meanwhile, the temperature at the outer parts of the glaze layer is relatively low, and that at the middle part is more uniform. From Figure 10e, we can see that when *h_g_* = 2.5 mm, the number of dense cracks increases, the blocks divided by the cracks in the middle become large, and the low temperature areas surround the dense cracks. Figure 10d shows that when *h_g_* = 3 mm, the crack density is similar to that when *h_g_* = 2.5 mm, but more through-cracks are generated in the middle. The temperature distribution on the model surface become relatively uniform.

**Figure 11:**
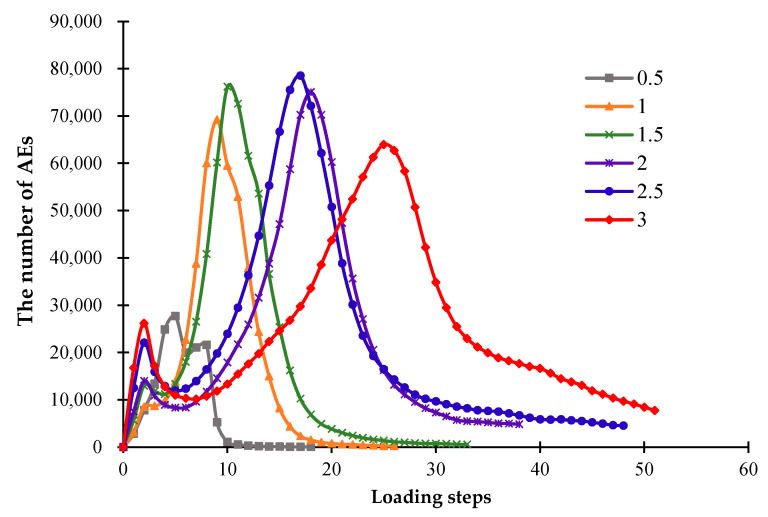
The acoustic emission curves affected by different glaze thicknesses.

Figure 11 shows that when the models with different thicknesses are cooled to the ambient temperature, the number of AEs display various responses. It can be seen that as the model thickness increases, more loading steps are required for cooling to the same level. When the model starts to cool down, there are many damaged areas on the ceramic surface due to rapid cooling from a high temperature, which may result in the first small peak of released AEs. As the cooling continues, the thinner model will produce a later second AE peak, and the model destruction will appear later. Namely, with the growth of the model thickness, the peak of released AEs appears relatively late, and the cooling time required to generate the macro failure of the model increases. This phenomenon occurs because greater temperature stresses are required to break the thicker model. Furthermore, when the thickness increases to a certain value, the number of cracks on the model surface will be largely reduced, and the destruction of the model becomes more difficult.

**Figure 12:**
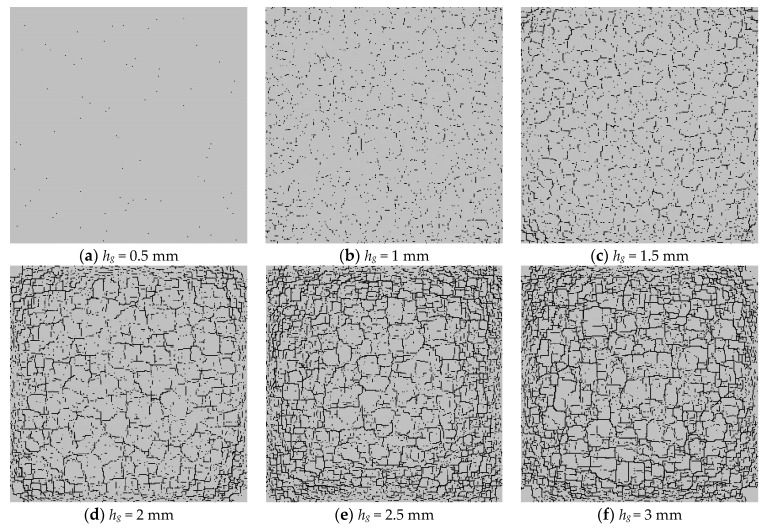
The glaze layer crack distribution affected by different glaze thicknesses.

**Figure 13:**
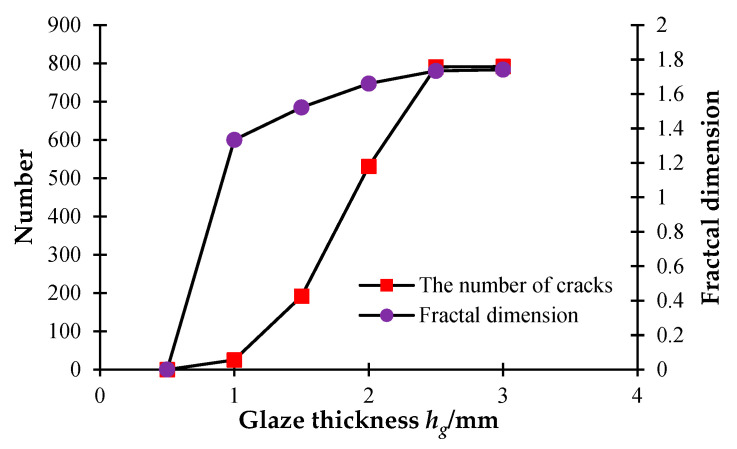
The number of cracks and fractal dimension curves under different glaze thickness.

Figure 12 shows that as the thickness of the glaze layer increases, cracks appear on the surface of the glaze layer when the model temperature reaches the ambient level. Figure 12a,b shows that when *h_g_* = 0.5–1 mm, there are a few microcracks scattered on the surface of the glaze layer, and no apparent through-cracks occur. Figure 12c indicates that when *h_g_* = 1.5 mm, some penetrating cracks begin to appear and spread over the model surface. Figure 12d–f demonstrates that as the layer thickness increases to 2 mm, more and more cracks spread over the model surface. Namely, as the model thickness increases, the number of cracks continues to rise. When the thickness of the glaze layer grows up to a certain level, the growth rate of the number of cracks becomes smaller. Additionally, with the thickness of the glaze layer increasing, the temperature stresses required to produce cracks becomes larger, meaning that the model is more difficult to be destroyed. From Figure 13, we can see that the fractal dimension shows a sudden increment when the glaze thickness rises from 0.5 mm to 1 mm, while after that, the fractal dimension rises slightly with the continuous increase in the glaze thickness.

### 3.4. The Influence of Coefficient of Thermal Expansion (CTE) of Glaze

The glaze and blank layers are two important components of ceramics, and their compatible deformation, controlled by the coefficient of thermal expansion (CTE) of a glaze, has a great influence on their mechanical responses. In order to understand the influence of the CTE of ceramics on the discrete fracture network, the following models are established. Clearly, the CTE of the blank is set to be 5 × 10^−6^/K and remains unchanged. But the CTE of the glaze layer changes, i.e., *α_g_* = 4.5 × 10^−6^/K, 5.5 × 10^−6^/K, 5.75 × 10^−6^/K, 6 × 10^−6^/K, 6.25 × 10^−6^/K, and 6.5 × 10^−6^/K, and *α_b_* = 5 × 10^−6^/K.

**Figure 14:**
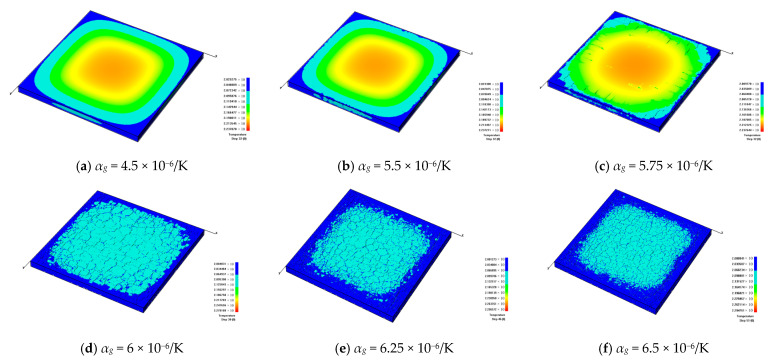
The development of cracks on the glaze surface and the temperature distribution when the glaze layers with different CTEs are cooled to the same degree: *h_g_* = 2 mm and *β* = 100 W/(m^2^·K).

Figure 14 shows the observed discrete fracture networks forming on the ceramic surfaces when the models are cooled to the same ambient temperature. From Figure 14a–f, we can see that a higher expansion coefficient of the glaze layer will lead to denser cracks on the surface of the glaze layer. Meanwhile, when the CTE of the glaze is smaller than that of the blank, the glaze layer shrinks less than does the blank layer during the cooling process. Furthermore, the high compressive stresses between the glaze and blank layers suppress the growth of tensile cracks near the ceramic surface. However, when the CTE of the glaze layer is greater than that of the blank, the shrinkage of the glaze layer becomes more severe than that of the blank body, resulting in high tensile stress concentrations near the ceramic surface. Therefore, many cracks are generated in the glaze layer.

**Figure 15:**
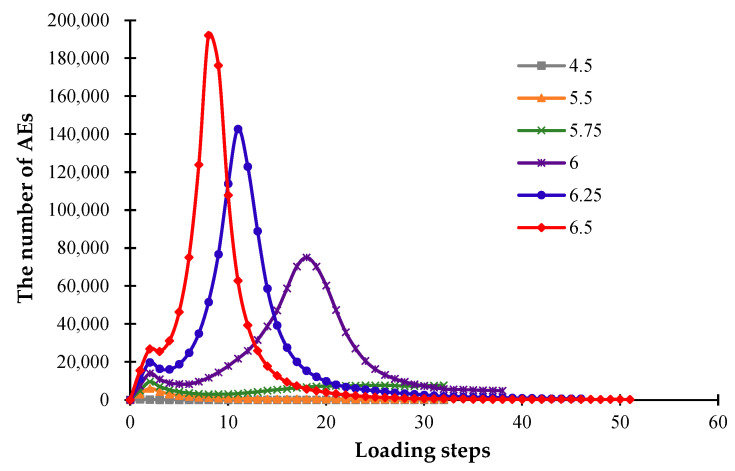
The acoustic emission curves affected by different glaze CTEs.

Figure 15 shows the changing trends of the AE events affected by different CTEs of the glaze, from which we can see that the significant difference between the expansion coefficients of the glaze and the blank layers will cause a greater degree of damage to the ceramics. Figure 15 also indicates that as the CTE of the glaze layer increases, the number of AE events also increases, and the peak of AE events occurs earlier.

**Figure 16:**
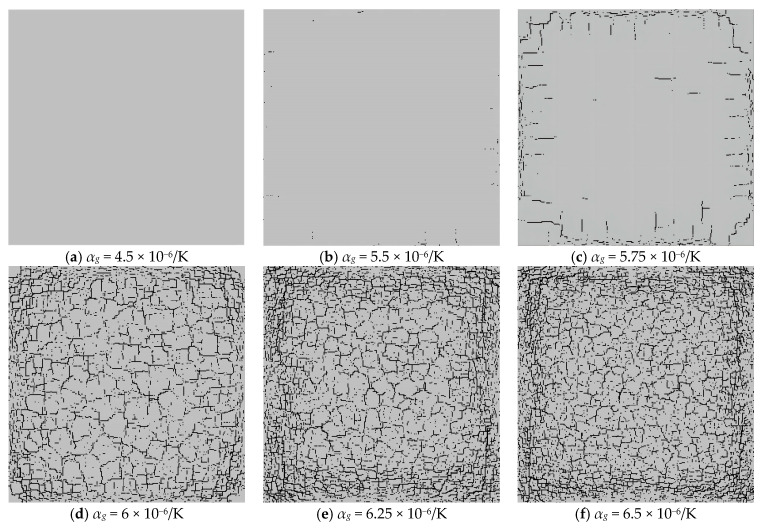
The glaze layer crack distribution affected by different glaze CTEs.

Figure 16 shows that the distribution and density of generated cracks change with the increase in the CTE of the glaze layer. Figure 16a indicates that when the CTE of the glaze layer is smaller than that of the blank, few cracks occur during the cooling process of the model. From Figure 16b, it is clear that when the CTE increases to 5.5 × 10^−6^/K, only a few microcracks appear, with little influence on the stability of the model. However, Figure 16c demonstrates that when the CTE rises to 5.75 × 10^−6^/K, some large-scale cracks began to appear at the outer parts of the model, but they do not extend to the center of the model. It is worth noting that in Figure 16d, when *α_g_* = 6 × 10^−6^/K, many cracks began to form on the model surface. Namely, as the CTE increases from 5.5 × 10^−6^/K to 6 × 10^−6^/K, the number of cracks rises quickly. Figure 16e,f shows that when *α_g_* increases to a certain value, the number of cracks remains nearly the same.

**Figure 17:**
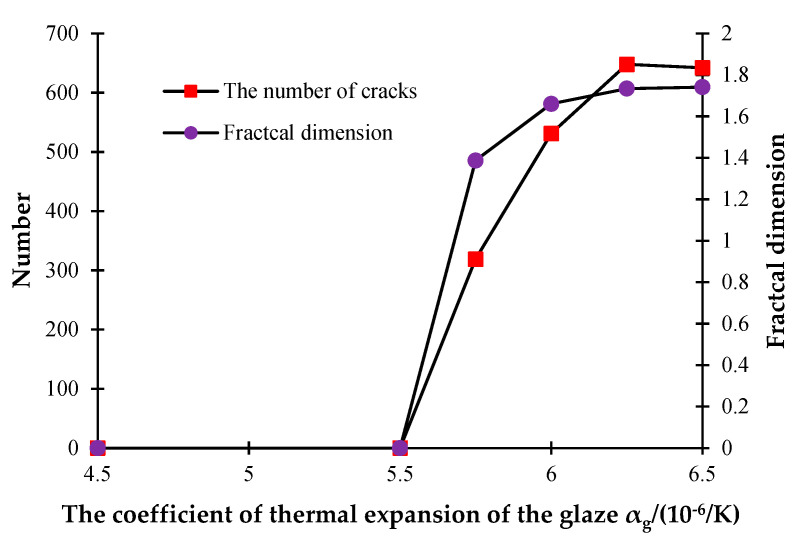
The number of cracks and fractal dimension curves under different glaze CTEs.

Figure 17 also illustrates the rapid growth of cracks when the CTE varies from 5.5 × 10^−6^/K to 6 × 10^−6^/K and the stability of cracks when the CTE reaches 6.25 × 10^−6^/K. These phenomena occur because the model will be destroyed earlier as the CTE increases, a large amount of heat will be lost after the formation of through-cracks, and it is therefore difficult to generate the concentrated temperature stresses on the model surface at the later stage. Moreover, Figure 17 shows that the fractal dimension increases quickly when the CTE rises from 5.5 × 10^−6^/K to 6 × 10^−6^/K, and the growth speed slows significantly when the CTE reaches 6.25 × 10^−6^/K.

## 4. Conclusions

In this study, the crack mode and the failure process of ceramic glaze during the cooling process are investigated, and the initiation and propagation of polygonal cracks are intuitively reproduced numerically. Furthermore, the formation mechanism of cracks inside ceramic glaze is analyzed. Meanwhile, the effects of different glaze layer thicknesses, expansion coefficients, and thermal conductivity coefficients of the glaze layer are discussed. The main conclusions can be drawn as follows:

(1) The cooling rate has a great influence on the formation of microcracks on a ceramic surface. Namely, a lower cooling rate will enhance the temperature difference in ceramics, leading to denser cracks. When the model temperature drops to essentially the temperature of surrounding environment, new cracks will not appear due to the small temperature difference, which means that the cracks on a ceramic surface become stable.
(2) A higher heat transfer coefficient will lead to a faster cooling speed and a small temperature difference on the ceramic surface. When all the ceramic models are cooled to the same degree, a higher heat transfer coefficient will result in a more uniform surface temperature. Furthermore, a higher heat transfer coefficient will cause a faster drop in the ceramic temperature, resulting in a smaller temperature difference inside the model and a smaller number of microcracks. When the heat transfer coefficient increases to a certain level, the model can be cooled quickly, and many damage zones will occur inside the model at the beginning of cooling. As the cooling process continues, the model with a higher heat transfer coefficient will experience the second massive failure sooner, the failure process will end earlier.
(3) As the thickness of the glaze layer increases, the distribution of cracks on a ceramic surface also changes. When the thickness of the glaze layer is much lower than that of the blank layer, only a few microcracks scatter on the surface of the glaze layer during the cooling process. As the thickness of the glaze layer increases, some through-cracks begin to appear. When the thicknesses of the glaze layer and the blank body becomes equal, cracks spread throughout the surface of ceramics. However, when the thickness of the glaze layer rises to a certain level, the number of cracks decreases and the growth of the cracks slows down, even showing a downward trend. Simultaneously, as the thickness of the glaze layer increases, the temperature stress required for producing cracks gradually rises, and the model becomes more difficult to destroy.
(4) When the CTE of the glaze layer is smaller than that of the blank body, no cracks will occur during the cooling process. When the CTE of the glaze layer is greater than that of the blank body, with the growth of the CTE of glaze layer, the number of cracks on the ceramic surface will gradually increase. However, when the CTE of the glaze layer increases to a certain level, the number of cracks starts to decrease. This phenomenon occurs because with the increasing CTE, the heat will be lost more and more quickly, and it is difficult for the temperature stress to accumulate on the ceramic surface.

## Figures and Tables

**Table 1:** Mechanical parameters used in numerical simulation.

Material	Elastic Modulus (*E*)/MPa	Compressive Strength (*σ_c_*)/MPa	Tensile Strength (*σ_t_*) /MPa	Poisson’s Ratio (*υ*)	Heterogeneity (*m*)	Thermal Capacity J/(m^3^·k)	Thermal Conductivity W/(m·k)
Glaze	61,240	450	90	0.24	8	2.1	1
Blank	100,000	800	160	0.3	5	2.1	1

## Data Availability

The datasets generated and/or analyzed during the current study are available from the corresponding author upon request. Moreover, for the references written in Chinese, the corresponding author will translate the relevant content into English upon request.

## References

[1] Bai T., Plollard D.D., Gao H. (2000). Explanation for fracture spacing in layered materials. Nature.

[2] Jitcharoen J., Padture N.P., Giannakopoulos A.E., Suresh S. (2005). Hertzian-crack suppression in ceramics with elastic-modulus-graded surfaces. J. Am. Ceram. Soc..

[3] Zok F.W., Spearing S.M. (1992). Matrix crack spacing in brittle matrix composites. Acta Metall. Mater..

[4] Vagaggini E., Domergue J.M., Evans A.G. (1995). Relationships between hysteresis measurements and the constituent properties of ceramic matrix composites: I, Theory. J. Am. Ceram. Soc..

[5] Daomergue J.M., Vagaggini E., Evans A.G. (1995). Relationships between hysteresis measurements and the constituent properties of ceramic matrix composites: II, Experimental studies on unidirectional materials. J. Am. Ceram. Soc..

[6] Li L.B. (2022). Interface wear effects in ceramic composite crack opening. J. Compos Mater..

[7] Sun Y.J., Singh R.N. (1998). The generation of multiple matrix cracking and fiber-matrix interfacial debonding in a glass composite. Acta Mater..

[8] Arslan O. (2003). Hertzian crack propagation in graded glass/ceramic composites. J. Am. Ceram..

[9] Weigel N., Dinkler D., Kroplin B.H. (2001). Micromechanically based continuum damage mechanics material laws for fiber-reinforced ceramics. Comput. Struct..

[10] Wang Y., Liu X., Xiong Y. (2022). Numerical simulation of zonal disintegration of surrounding rock in the deep-buried chamber. Deep Undergr. Sci. Eng..

[11] Li K., Wang D.L., Chen H., Guo L.C. (2014). Normalized evaluation of thermal shock resistance for ceramic materials. J. Adv. Ceram..

[12] Wu X.F., Jiang C.P., Song F., Li J., Shao Y.F., Xu X.H., Yan P. (2015). Size effect of thermal shock crack patterns in ceramics and numerical predictions. J. Eur. Ceram. Soc..

[13] Jiang C.P., Wu X.F., Li J., Song F., Shao Y.F., Xu X.H., Yan P. (2012). A study of the mechanism of formation and numerical simulations of crack patterns in ceramics subjected to thermal shock. Acta Mater..

[14] Liu Y.X., Wu X.F., Guo Q.K., Jiang C.P., Song F., Li J. (2015). Experiments and numerical simulations of thermal shock crack patterns in thin circular ceramic specimens. Ceram. Int..

[15] Hong C., Shen H., Xu Q., Lu X.L., Cao C. (2014). Factors influencing transparent crackle glaze. China Ceram. Ind..

[16] Dong Y.L., Wang W.M. (2004). Progress of investigation on thermal shock resistance of ceramic materials. J. Adv. Ceram..

[17] Li W.J., Zhang Y., Zhang X.H., Hong C.Q., Han W.B. (2009). Thermal shock behavior of ZrB_2_-SiC Ultra-high temperature ceramics with addition of zirconia. J. Alloys Compd..

[18] Gyak K.W., Vishwakarma N.K., Hwang Y.H., Kim J., Yun H.S., Kim D.P. (2019). 3D-printed monolithic SiCN ceramic microreactors from a photocurable preceramic resin for the high temperature ammonia cracking process. React. Chem. Eng..

[19] Duan W.J., Jia D.C., Cai D.L., Niu B., Yang H.L., Yang Z.H., Zhou Y. (2021). Thermal shock damage assessment of a BNW/SiO_2_ ceramic: Insight from temperature-dependent material properties. Ceram. Int..

[20] Qi F., Meng S.H., Song F., Guo H., Xu X.H., Shao Y.F., Chen Y. (2019). Fractal characterization of ceramic crack patterns after thermal shocks. J. Am. Ceram. Soc..

[21] Zuo C., Li Q., Wang Q., Li Y., Li L., Wei J., Shao Y., Song F. (2022). Effect of material parameters on thermal shock crack of ceramics calculated by phase-field method. J. Am. Ceram. Soc..

[22] Osada T., Watabe A., Yamamoto J., Brouwer J.C., Kwakernaak C., Ozaki S., van der Zwaag S., Sloof W.G. (2020). Full strength and toughness recovery after repeated cracking and healing in bone-like high temperature ceramics. Sci. Rep..

[23] Ma T.C. (2011). Ceramic Technology.

[24] Zhou J.E., Liang D., Li Q.J., Zhang M.L., Wu J.M. (2011). Study on the formation mechanism and preparation of ice crackle celadon. J. Synth. Cryst..

[25] Wei A., Liu Z., Zhang F., Ma M., Chen G., Li Y. (2020). Thermal expansion coefficient tailoring of LAS glass-ceramic for anodic bondable low temperature co-fired ceramic application. Ceram. Int..

[26] Roy S., Nagel A., Weidenmann K.A. (2020). Anisotropic thermal expansion behavior of an interpenetrating metal/ceramic composite. Thermochim. Acta.

[27] Wang Q., Li F. (2021). Controllable thermal expansion in ferroelectric ceramics through two-dimensional periodically orthogonal poling. Scr. Mater..

[28] Fan X., Ma X., Dang X., Xue J., Ye F., Zhao D., Cheng L. (2020). In-plane thermal expansion behavior of dense ceramic matrix composites containing SiBC matrix. J. Eur. Ceram. Soc..

[29] Tavangarian F., Hui D., Li G. (2018). Crack-healing in ceramics. Compos. Part B Eng..

[30] Junior G.S., Ferreira J., Millán-Arias C., Daniel R., Junior A.C., Fernandes B.J.T. (2021). Ceramic cracks segmentation with deep learning. Appl. Sci..

[31] Shao C.X., Guo H., Meng S.H., Shao Y.F., Wang S.X., Xie S.J., Qi F. (2022). Characterization of ceramic thermal shock cracks based on the multifractal spectrum. Fractal Fract..

[32] Zhu W.W., Lei G., He X.P., Patzek T.W., Wang M. (2022). Fractal and multifractal characterization of stochastic fracture networks and real outcrops. J. Struct. Geol..

[33] Wang Y., Gong B., Tang C. (2022). Numerical investigation on anisotropy and shape effect of mechanical properties of columnar jointed basalts containing transverse joints. Rock Mech. Rock Eng..

[34] Gong B., Liang Z.Z., Liu X.X. (2022). Nonlinear deformation and failure characteristics of horseshoe-shaped tunnel under varying principal stress direction. Arab. J. Geosci..

[35] Wang Y., Gong B., Zhang Y., Yang X., Tang C. (2022). Progressive fracture behavior and acoustic emission release of CJBs affected by joint distance ratio. Mathematics.

[36] Liang Z.Z., Xiao D.K., Li C.C., Wu X.K., Gong B. (2014). Numerical study on strength and failure modes of rock mass with discontinuous joints. Chin. J. Geotech. Eng..

[37] Tang S.B. (2009). Mesoscopic Numerical Model of Thermo-Hydro Cracking Process Concrete. Ph.D. Thesis.

[38] Li G., Wang K., Gong B., Tao Z.G., Du K. (2021). A multi-temporal series high-accuracy numerical manifold method for tran-sient thermoelastic fracture problems. Int. J. Solids Struct..

[39] Chen T.T., Foulger G.R., Tang C.A., Mathias S.A., Gong B. (2022). Numerical investigation on origin and evolution of polygonal cracks on rock surfaces. Eng. Geol..

[40] Tang C.S., Cui Y.J., Tang A.M., Shi B. (2010). Experiment evidence on the temperature dependence of desiccation cracking behavior of clayey soils. Eng. Geol..

[41] Tang C.S., Shi B., Liu C., Zhao L., Wang B. (2008). Influencing factors of geometrical structure of surface shrinkage cracks in clayey soils. Eng. Geol..

